# Identification of HNF4A Mutation p.T130I and HNF1A Mutations p.I27L and p.S487N in a Han Chinese Family with Early-Onset Maternally Inherited Type 2 Diabetes

**DOI:** 10.1155/2016/3582616

**Published:** 2016-02-11

**Authors:** Ying Yang, Tai-Cheng Zhou, Yong-Ying Liu, Xiao Li, Wen-Xue Wang, David M. Irwin, Ya-Ping Zhang

**Affiliations:** ^1^Department of Endocrinology, The Second People's Hospital of Yunnan Province, Kunming, Yunnan 650021, China; ^2^Laboratory for Conservation and Utilization of Bio-Resources, Yunnan University, Kunming, Yunnan 650091, China; ^3^The Central Laboratory of the Second People's Hospital of Yunnan Province, Kunming, Yunnan 650021, China; ^4^Laboratory of Biochemistry and Molecular Biology, Yunnan University, Kunming, Yunnan 650091, China; ^5^State Key Laboratory of Genetic Resources and Evolution, Kunming Institute of Zoology, Chinese Academy of Sciences, Kunming, Yunnan 650223, China; ^6^Department of Laboratory Medicine and Pathobiology, University of Toronto, Toronto, ON, Canada M5S 2E8

## Abstract

Maturity-onset diabetes of the young (MODY) is characterized by the onset of diabetes before the age of 25 years, positive family history, high genetic predisposition, monogenic mutations, and an autosomal dominant mode of inheritance. Here, we aimed to investigate the mutations and to characterize the phenotypes of a Han Chinese family with early-onset maternally inherited type 2 diabetes. Detailed clinical assessments and genetic screening for mutations in the* HNF4α*,* GCK*,* HNF-1α*,* IPF-1*,* HNF1β*, and* NEUROD1 *genes were carried out in this family. One HNF4A mutation (p.T130I) and two HNF1A polymorphisms (p.I27L and p.S487N) were identified. Mutation p.T130I was associated with both early-onset and late-onset diabetes and caused downregulated* HNF4A* expression, whereas HNF1A polymorphisms p.I27L and p.S487N were associated with the age of diagnosis of diabetes. We demonstrated that mutation p.T130I in HNF4A was pathogenic as were the predicted polymorphisms p.I27L and p.S487N in HNF1A by genetic and functional analysis. Our results show that mutations in* HNF4A *and* HNF1A* genes might account for this early-onset inherited type 2 diabetes.

## 1. Introduction

Type 2 diabetes mellitus (T2DM) is a genetically complex metabolic disorder characterized by hyperglycemia that leads to serious complications damaging the health of people. Maturity-onset diabetes of the young (MODY) is characterized by an early age of onset, positive family history, and a monogenic autosomal dominant pattern of inheritance [1]. Mutations causing MODY have been described in at least 13 genes:* HNF4A*,* GCK*,* HNF1A*,* IPF1*,* HNF1B*,* NEUROD1*,* KLF11*,* CEL*,* PAX4*,* INS*,* BLK*,* ABCC8*, and* KCNJ11* [2–4]. Among these genes, six have been identified as important factors for MODY in many populations. These 6 genes encode hepatocyte nuclear factor 4*α* (HNF4A; MODY1) [5], glucokinase (GCK; MODY2) [6, 7], HNF1*α* (HNF1A; MODY3) [8], insulin promoter factor 1 (IPF1; MODY4) [9], HNF1*β* (HNF1*β*; MODY5) [10], and neurogenic differentiation factor 1/BETA2 (NEUROD1; MODY6) [11]. All of the proteins encoded by these 6 genes, except glucokinase, are transcription factors. The prevalence of the MODY subtypes varies in different areas. Globally, MODY2 and MODY3 mutations constitute approximately 70% of MODY cases [12], while the other four types of MODY are more rarely found. For Asians, however, only 7.5%–10.3% of patients have mutations in MODY2 and MODY3 [13], and MODY with unknown cause (MODYX) accounts for 80–90% of clinical MODY cases [14].

There are, however, few reports concerning MODY gene mutations in Han Chinese families; thus additional mutational profiling is needed [13, 14]. In this study, we screened 6 MODY genes (*HNF4A*,* GCK*,* HNF1A*,* IPF1*,* HNF1B*, and* NEUROD1*) for mutations in a Han Chinese family with suspected early-onset maternally inherited type 2 diabetes. Within this family, a mutation was found within HNF4A (p.T130I) and two polymorphisms (p.I27L and p.S487N) in HNF1A were identified. Functional analysis demonstrated that the HNF4A gene mutation T130I causes reduced expression in HeLa cells and thus affects protein function, whereas the HNF1A polymorphisms p.I27L and p.S487N may be associated with the age of diagnosis of diabetes. The three mutations described here appear to be associated with early-onset maternally inherited type 2 diabetes and yield differing clinical characteristics.

## 2. Materials and Methods

### 2.1. Patients

**Figure 1:**
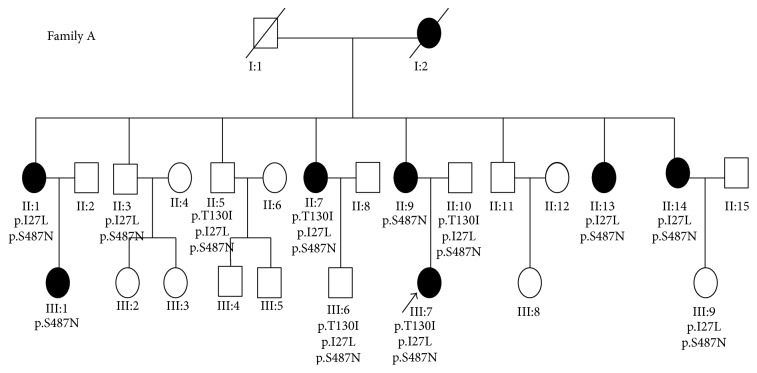
Pedigree of the family examined in this study.

A Han Chinese type 2 diabetes family from Yunnan, Southwest China, was enrolled for this study (Figure 1). This case was diagnosed in the Second People's Hospital of Yunnan Province, Kunming, China. We recruited members from three generations of this family, including 26 subjects with 13 maternal members and 13 nonmaternal members. Diagnosis and classification of diabetes are based on clinical features, laboratory data, and the guidelines of the Expert Committee Report of the American Diabetes Association. Informed consents conforming to the tenets of the Declaration of Helsinki and following the guidance of sample collection of Human Genetic Disease (863 program) by the Ministry of Public Health of China were obtained from each participant prior to the study. The institutional review boards of the Second People's Hospital of Yunnan Province and Yunnan University approved this study.

### 2.2. DNA Extraction, MODY Genes Sequencing, and Data Quality Control

Genomic DNA was isolated from whole blood using the standard phenol/chloroform method. All exons and flanking intron regions of the 6 MODY genes were amplified from the genomic DNA samples by the polymerase chain reaction (PCR) according to published methods [15]. Primer pairs and PCR conditions are listed in Table S1 in Supplementary Material available online at http://dx.doi.org/10.1155/2016/3582616. PCR products were purified on spin columns (Watson Biotechnologies Inc., Shanghai, China) and directly sequenced by the PCR or sequencing primers described above using the BigDye Terminator v3.1 Cycle Sequencing Kit (Applied Biosystems) performed on a 3730 DNA analyzer according to the manufacturer's manual. To ensure the quality of the DNA sequences, PCR products were sequenced at least twice in both directions. DNA sequences were edited using DNASTAR's SeqMan software (DNASTAR Inc., Madison, WI, USA). Changes in the sequence were checked against published polymorphisms and mutations from the Human Genome Variation Society (HGVS, http://www.HGVS.org).

### 2.3. Sequence Analysis

Functional amino acid motifs were predicted using the MotifScan program in the PROSITE database of protein families and domains (http://www.expasy.org/prosite). Transmembrane and surface regions were verified using HMMTOP (v.2.0) (http://www.enzim.hu/hmmtop/). Amino acid substitutions were examined for their possible effects on protein function with the programs SIFT (http://sift-dna.org) and PolyPhen-2 (http://genetics.bwh.harvard.edu/pph2/). Tertiary structures of proteins were predicted with the I-TASSER suite (Iterative Threading ASSEmbly Refinement) [16].

### 2.4. Plasmid Constructs

Here, we used* HNF4A1* (GenBank accession number: NM_178849.2) not* HNF4A2* as the former is also highly expressed in liver but has rarely been examined [17]. Full-length human wild type* HNF4A1* cDNA was cloned into pCDH-CMV-MCS-EF1-Puro (System Biosciences, Mountain View, CA, USA) to create pCDH/HNF4A (HNF4A-WT). The mutant pCDH/HNF4A-T130I (HNF4A-T130I) was generated by PCR-based mutagenesis using HNF4A-WT as a template and primers containing the nucleotide change.

Luciferase assay reporter constructs using pGL3 Basic vector (Promega) were also prepared. The human HNF1A gene promoter (–129/+196) constructs were subcloned into the pGL3 Basic vector using a PCR strategy or by restriction enzyme sites [18]. All vector constructs were verified by DNA sequencing on both strands.

### 2.5. Cell Culture and Transfection

HeLa and Hep-G2 cells were cultured in DMEM supplemented with 10% fetal bovine serum with 1% nonessential amino acids (Nacalai Tesque) at 37°C and 5% CO_2_. Upon reaching 80–90% confluence, HeLa and Hep-G2 cells were plated in 96-well plates at a density of 3 × 10^4^ and 1 × 10^5^ cells per well, respectively. Subsequently, cells were incubated for 24 h prior to transfection. For transfecting HeLa and Hep-G2 cells, Lipofectamine 2000 (Invitrogen) was used with 0.4 *μ*g of HNF4A-WT, HNF4A-T130I, or empty vector (total of 0.8 *μ*g), 0.2 *μ*g reporter construct, and 0.05 *μ*g phRL-TK, according to the manufacturer's instructions (Table 1).

### 2.6. Western Blot Analysis

All reagents were purchased from Sigma if not indicated otherwise. Forty-eight hours after transfection, HeLa cells in the 6-well plates were washed twice with phosphate-buffered saline (PBS) and scraped into 200 *μ*L/well of lysis buffer [150 mM NaCl, 50 mM Tris-HCl (pH 7.5), 1% NP40, 0.25% sodium deoxycholate, and 1 mM EDTA with one tablet of protease inhibitor cocktail tablet (Complete Mini, Roche) per 10 mL], sonicated for 2-3 s, and then centrifuged. Supernatant (5 *μ*g of protein) was fractionated on a 10% sodium dodecyl sulfate-polyacrylamide gel and blotted to a nitrocellulose membrane (TEFCO). Membranes were blocked with PBS containing 5% skimmed milk and then incubated overnight at 4°C with rabbit monoclonal anti-human HNF4*α* antibody (1 : 1,000; Cell Signaling Technology, Danvers, MA, USA). After washing, membranes were reblocked and incubated for 2 h at room temperature in the presence of horseradish peroxidase-linked donkey anti-rabbit IgG antibody (1 : 10,000; GE Healthcare, Little Chalfont, UK) and developed using the SuperSignal West Dura Extended Duration Substrate (Thermo Scientific, Waltham, MA, USA). As an internal control, *β*-actin was detected with a mouse monoclonal anti-*β*-actin antibody (1: 10,000).

### 2.7. Luciferase Reporter Assay

Following a 48-hour incubation, the transcriptional activities of the wild type and mutant HNF4A proteins were measured by the Dual-Glo Luciferase Reporter Assay System (Promega). All transfections were carried out in triplicate. We performed six independent experiments.

### 2.8. Statistical Analysis

Data are expressed as mean ± SD and analyzed with unpaired *t*-tests using JMP software version 8.0.1 (SAS Institute Inc., Cary, NC, USA). *p* value < 0.01 was considered significant.

## 3. Results 

### 3.1. Patients

We evaluated the clinical phenotype and clinical assessments scores for individuals from the family in this study (Table S2). Affected individuals from this family had histories of hyperglycemia (plasma glucose > 6 mmol/L, fasting) with normal fasting insulin levels. The proband was diagnosed with diabetic ketosis when she was 13 years old and experienced weakness, polydipsia, polyuria, and the loss of 5 kilograms in weight during a 3-month stay in Beijing Hospital. After 2 years without any medicine treatment and only rare occurrences of blood glucose monitoring, the proband visited our hospital for blurred vision. At that time her blood glucose levels ranged from 8 to 15 mmol/L. A fundus fluorescein angiography showed that she suffered from nonproliferative retinopathy in her right eye and proliferative retinopathy in left eye (Figure S1).

A total of 25 additional family members that covered 3 generations and included 12 maternal and 13 nonmaternal members were examined (Figure 1). A total of 7 additional members of this family had previously been diagnosed with diabetes. The average age of onset of diabetes in this family was 37 ± 5 years old, with a penetrance within this family of 30.77% (8/26).

### 3.2. Screening for MODY Mutations

**Figure 2:**
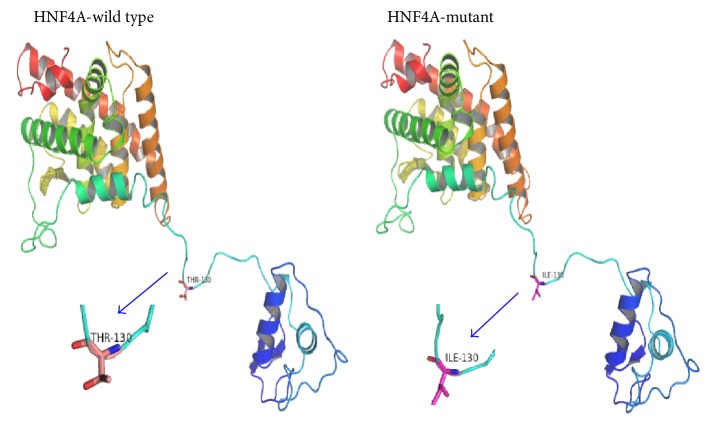
Homology modeling of the HNF4A protein with and without p.T130I.

**Figure 3:**
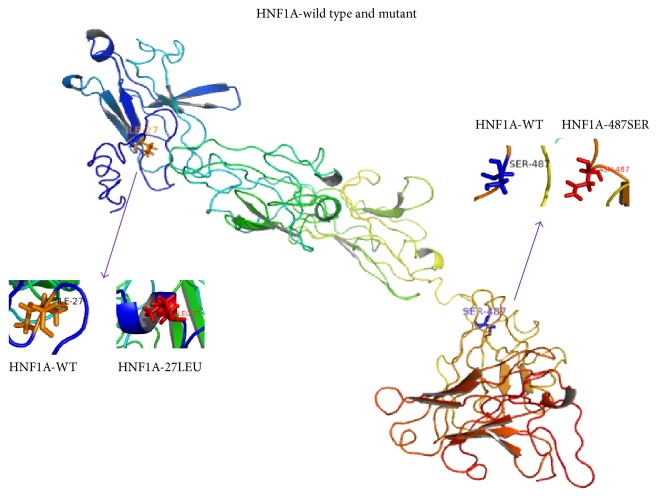
Homology modeling of the HNF1A protein with and without p.I27L and p.S487N.

**Figure 4:**
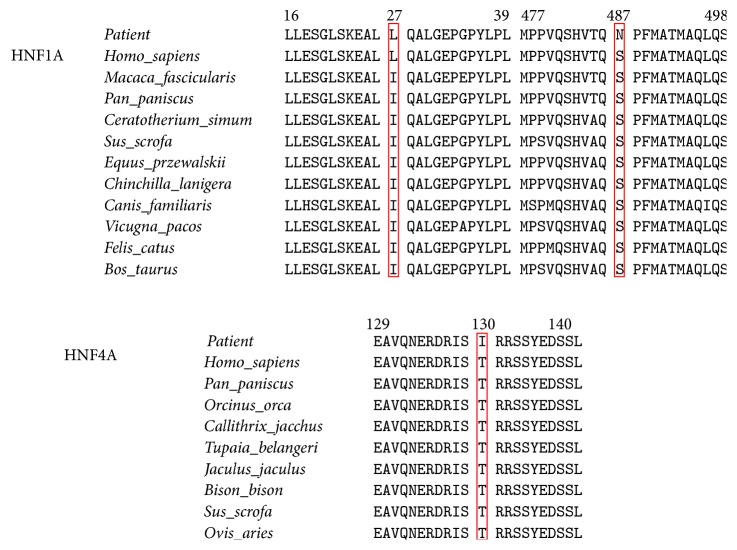
Evolutionary conservation analysis for mutations p.T130I in HNF4A and p.I27L and p.S487N in HNF1A.

All exons for all six MODY genes were amplified and sequenced from the proband. A total of three missense mutations were identified in these sequences compared to wild type sequences, two in* HNF1A* (p.I27L and p.S487N) and one in* HNF4A* (p.T130I) (Table S2 and Figure S2). No mutations in any of the other MODY genes were detected. The mutation p.T130I in exon 7 of* HNF4A* maps to a protein kinase C phosphorylation site in the protein that is located on a cytoplasmic loop (Figure 2). The mutation p.I27L in exon 1 of* HNF1A* is located between an *α*-helix on the surface and a loop in the protein structure, while mutation p.S487N in exon 5 is located in a cytoplasmic loop (Figure 3). An evolutionary conservation analysis revealed that mutations p.T130I in HNF4A and p.S487N in HNF1A occur at highly conserved positions in the protein sequences and do not involve similar amino acids, while mutation p.I27L in HNF1A is a conservative change at a relatively conserved isoleucine (Figure 4). Results from SIFT, PolyPhen-2, and Mutation Taster predict a probable damaging effect and disease causing effect for the p.T130I replacement in HNF4A, but mutations p.I27L and p.S487N in HNF1A were predicted to be tolerated.

### 3.3. Functional Properties of the HNF4A-T130I Substitution

**Figure 5:**
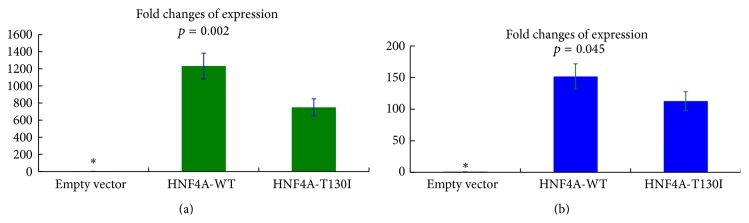
Expression levels of wild type and p.T130I HNF4A in transfected cells. *∗* indicates the baseline value. HeLa (a) and Hep-G2 (b) cells were transfected.

**Figure 6:**
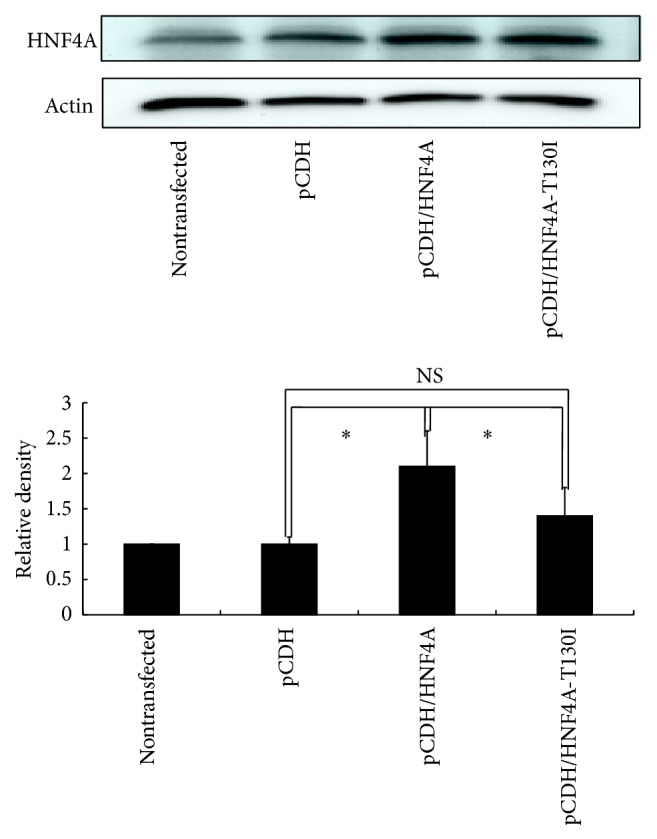
Western blot analysis of wild type and p.T130I HNF4A. HeLa cells were transfected with the different vectors as indicated. Nontransfected, pCDH, pCDH/HNF4A, and pCDH/HNF4A-T130I correspond to 1, 2, 3, and 4 in the transfection studies. ^*∗*^ *p* < 0.05 (*n* = 3).

Since the p.T130I replacement in HNF4A was predicted to have a damaging effect, we investigated the functional consequences of this substitution. For this we transfected HeLa and Hep-G2 cells with HNF4A-WT and HNF4A-T130I expression vectors. Real-Time PCR was used to assess the expression patterns of HNF4A-WT or HNF4A-T130I in HeLa and Hep-G2 cell lines. Gene expression levels of cells transfected with the empty vector were set as baseline (marked with *∗* in Figure 5) and expression levels of the WT and mutant HNF4A were compared. In Hep-G2 cells, expression of HNF4A-WT was more than 151-fold higher than the baseline value, while HNF4A-T130I was 112-fold greater. In HeLa cells, expression of HNF4A-WT was more than 1232-fold higher than baseline, whereas expression of HNF4A-T130I was 749-fold greater (Figure 5). The HeLa results were verified by Western blot analysis (Figure 6), where the abundance of HNF4A-WT was approximately twice that of HNF4A-T130I. The transactivation abilities of HNF4A-WT and HNF4A-T130I were assessed using a luciferase reporter gene assay. No significant difference in luciferase activities was detected between HNF4A-WT and HNF4A-T130I transfected cells when pGL3-HNF1A promoter reporter constructs were used in HeLa and Hep-G2 cells (data not shown). In Hep-G2 cells, the transfection efficiency was low (less than 20%), and these cells failed to demonstrate a significant increase in luciferase activity after transfection with HNF4A.

## 4. Discussion

This family examined here appears to have maternally inherited diabetes, with a maternally transmitted penetrance (61.54%, 8/13) similar to other Chinese families; thus we first examined their complete mtDNA genomes [19]. As this diabetic family has an early age of onset and a positive family history, we followed up this study by examining their MODY genes by sequencing.

*HNF4A* is one of the most commonly mutated and important causative genes for MODY, with more than 33 mutations being reported [20]. Mutation p.T130I (c.389C>T) has previously been described in European patients with MODY [21–23] and was identified here in our study. The p.T130I mutation changes a threonine residue that is located between the DNA binding domain and the ligand binding domain, a region of the protein implicated in dimerization and DNA binding [24, 25], and the sequence of this region is conserved across many species (Figure 4). Interestingly, threonine (T130) residue is located within two putative protein kinase C phosphorylation sites (PKC phosphorylation sites, 129–131: STR and 130–132: TRR), while the isoleucine (I130) mutation would only allow one of these PKC phosphorylation sites (129–131: SIR) to be retained. The loss of a PKC phosphorylation site in HNF4A might affect the activation efficiency of HNF4A [26, 27]. To further investigate the potential impact of this mutation in HNF4A, we used* HNF4A1* (GenBank accession number: NM_178849.2), which had not previously been used to test the effect of this mutation but has an expression profile similar to* HNF4A2* [28]. Expression of HNF4A-T130I was significantly lower than that for HNF4A-WT based on Real-Time PCR and Western blot analyses. Moreover, a dominant negative effect seen in the transfection experiment, combined with the genetic data, strongly suggests that this mutation is diabetogenic. These results are consistent with previous research [29]. However, the reporter promoter construct containing the native HNF1A promoter demonstrated minimal transcriptional activity in our hands. Though specifically expressed in Hep-G2 cells, less is known about the transcriptional activity of HNF4*α*1 compared to the better characterized HNF4*α*2 [30]. This difference may explain the difficulty in obtaining statistically significant data using the HNF1A promoter. The low transfection efficiency in HeLa and Hep-G2 cells may also be another reason why we could not obtain statistically significant data in these cells.

The p.T130I HNF4A mutation is associated not only with MODY but also with the development of the common late-onset form of type 2 diabetes [31]. This may explain why some of the patients in our study with the p.T130I mutation had late onset. We note that one individual, III:6, in this family carries the p.T130I mutation but does not yet have type 2 diabetes; however, he is less than 40 years of age. The fact that Thr 130 in HNF4A is highly conserved between species and the high penetrance observed in the family members makes it highly probable that the p.T130I mutation in HNF4A is responsible for DM in this family.

*HNF1A* is also a common cause of MODY in many populations, with more than 193 mutations being described [32]. Here, we identified the p.I27L and p.S487N polymorphisms in HNF1A in a diabetes family. Polymorphism p.I27L is associated with an increased risk of type 2 diabetes, particularly in the elderly (age > 60 years) and overweight, while polymorphisms p.I27L and p.S487N together are associated with an earlier age of diagnosis [33–35]. In our family we observed that an individual carrying both the p.I27L and the p.S487N polymorphism (III:7) had an earlier age of diagnosis at 19 years of age.

Our evolutionary analyses suggested that the polymorphisms p.I27L and p.S487N occur at conserved sites and are located to the dimerization and transactivation domains of HNF1A, respectively. Interestingly, according to our predictions, the p.I27L polymorphism changes the tertiary structure of the protein from a loop to an *α*-helix at this site (Figure 3). This mutation thus potentially affects the ability of HNF1A to form dimers. Although p.I27L and p.S487N have been identified as polymorphisms that might not be associated with DM by some researchers [36], our evolutionary conservation analysis and protein tertiary structure predictions indicate that these two missense mutations, especially p.I27L, still might play role in the development of DM in the family examined in our study. The pathogenic mechanism of p.I27L and p.S487N is unknown and needs further functional investigation.

## Supplementary Material

Figure S1 A fundus fluorescein angiography showed that she suffered from nonproliferative retinopathy in her right eye and proliferative retinopathy in left eye. Nonproliferative retinopathy. (A)With intraretinal hemorrhage dots (large arrow) and microaneurysms (small arrow). (B) Fluorescein angiography of the eye shown in A. Leakage from capillary drop-out (open arrow), microaneurysms (small arrow) are seen as multiple dots of hyperfluorescemce, and capillary nonperfusion (large arrow) and the dot hemorrhage do not fluoresce; Proliferative retinopathy. (C) With viteous hemorrhage, intraretinal hemorrhage (small arrow), optic disk edema (larger arrow), and macular edema (open arrow). (D) . Early phase fluorescein angiography shows microaneurysms (small arrow).
Figure S2 Sequencing chromatogram for mutations p.T130I(c.389C>T) in HNF4A, and p.I27L(c.79A>C) and p.S487N(c.1460G>A) in HNF1A.
Table S1 Primers for amplifying and sequencing MODY genes.
Table S2 Clinical information on 12 members of family A.

## Figures and Tables

**Table 1:** HeLa cells were transfected with a combination of different vectors.

HeLa	1	2	3	4
Empty pCDH-vector	−	++	+	+
HNF4A-WT vector	−	−	+	−
HNF4A-T130I vector	−	−	−	+
PGL-HNF1A reporter vector	+	+	+	+
TK	+	+	+	+

Note: 4 *μ*g HNF4A-WT, HNF4A-T130I vector, or empty pCDH-vector (total of 8 *μ*g), 3 *μ*g reporter construct were transfected and 0.2 *μ*g TK was used to control transfection efficiency.
