# Supplementary material for: Identification of HNF4A Mutation p.T130I and HNF1A Mutations p.I27L and p.S487N in a Han Chinese Family with Early-Onset Maternally Inherited Type 2 Diabetes
Source: J Diabetes Res. 2016 Feb 11;2016:3582616. doi: 10.1155/2016/3582616 (PMC4766352; doi:10.1155/2016/3582616)
Supplement: Supplementary file 1 — Figure S1 A fundus fluorescein angiography showed that she suffered from nonproliferative retinopathy in her right eye and proliferative retinopathy in left eye. Nonproliferative retinopathy. (A)With intraretinal hemorrhage dots (large arrow) and microaneurysms (small arrow). (B) Fluorescein angiography of the eye shown in A. Leakage from capillary drop-out (open arrow), microaneurysms (small arrow) are seen as multiple dots of hyperfluorescemce, and capillary nonperfusion (large arrow) and the dot hemorrhage do not fluoresce; Proliferative retinopathy. (C) With viteous hemorrhage, intraretinal hemorrhage (small arrow), optic disk edema (larger arrow), and macular edema (open arrow). (D) . Early phase fluorescein angiography shows microaneurysms (small arrow). Figure S2 Sequencing chromatogram for mutations p.T130I(c.389C>T) in HNF4A, and p.I27L(c.79A>C) and p.S487N(c.1460G>A) in HNF1A. Table S1 Primers for amplifying and sequencing MODY genes. Table S2 Clinical information on 12 members of family A. [file 3582616.f1.zip › Supporting Information.docx]

**Supporting Information**

**Figure S1** A fundus fluorescein angiography showed that she suffered from nonproliferative retinopathy in her right eye and proliferative retinopathy in left eye.

Nonproliferative retinopathy. (A)With intraretinal hemorrhage dots (large arrow) and microaneurysms (small arrow). (B) Fluorescein angiography of the eye shown in A. Leakage from capillary drop-out (open arrow), microaneurysms (small arrow) are seen as multiple dots of hyperfluorescemce, and capillary nonperfusion (large arrow) and the dot hemorrhage do not fluoresce; Proliferative retinopathy. (C) With viteous hemorrhage, intraretinal hemorrhage (small arrow), optic disk edema (larger arrow), and macular edema (open arrow). (D) . Early phase fluorescein angiography shows microaneurysms (small arrow).

**Figure S2** Sequencing chromatogram for mutations p.T130I(c.389C>T) in

HNF4A, and p.I27L(c.79A>C) and p.S487N(c.1460G>A) in HNF1A.

**Table S1** Primers for amplifying and sequencing MODY genes;

**Table S2** Clinical information on 12 members of family A.
